# Dietary Inclusion of Black Soldier Fly (*Hermetia Illucens*) Larvae Meal and Paste Improved Gut Health but Had Minor Effects on Skin Mucus Proteome and Immune Response in Atlantic Salmon (*Salmo Salar*)

**DOI:** 10.3389/fimmu.2021.599530

**Published:** 2021-02-25

**Authors:** Pabodha Weththasinghe, Leidy Lagos, Marcos Cortés, Jon Øvrum Hansen, Margareth Øverland

**Affiliations:** ^1^ Department of Animal and Aquacultural Sciences, Faculty of Biosciences, Norwegian University of Life Sciences, Ås, Norway; ^2^ Laboratory of Immunology, Centre of Aquatic Biotechnology, Department of Biology, Faculty of Chemistry and Biology, University of Santiago of Chile, Santiago, Chile

**Keywords:** black soldier fly larvae, meal, paste, Atlantic salmon, gut health, plasma biochemical parameters, immune response, skin mucus proteome

## Abstract

The present study investigated effects of dietary inclusion of black soldier fly larvae (BSFL) (*Hermetia illucens*) meal and paste on gut health, plasma biochemical parameters, immune response and skin mucus proteome in pre-smolt Atlantic salmon (*Salmo salar*). The seven-week experiment consisted of seven experimental diets: a control diet based on fishmeal and plant protein (Control-1); three BSFL meal diets, substituting 6.25% (6.25IM), 12.5% (12.5IM) and 25% (25IM) of protein; two BSFL paste diets, substituting 3.7% (3.7IP) and 6.7% (6.7IP) of protein and an extra control diet with 0.88% of formic acid (Control-2). The 6.25IM diet reduced enterocyte steatosis in pyloric caeca, improved distal intestine histology, and reduced IgM in distal intestine. The fish fed 12.5IM diet reduced enterocyte steatosis in pyloric caeca, improved distal intestine histology, had a higher plasma lysozyme content compared to 6.25IM, and tend to increase phagocytic activity in head-kidney macrophages-like cells. On the other hand, 25IM diet improved distal intestine histology, but showed mild-moderate enterocyte steatosis in pyloric caeca, increased IFNγ and reduced IgM in distal intestine. In the case of BSFL paste diets, 3.7IP diet caused mild inflammatory changes in distal intestine, although it reduced enterocyte steatosis in pyloric caeca. The 6.7IP diet reduced enterocyte steatosis in pyloric caeca and improved distal intestine histology. Increasing level of BSFL meal in the diet linearly decreased plasma C-reactive protein, whereas increasing level of BSFL paste linearly increased plasma antioxidant capacity. Dietary inclusion of BSFL meal and paste had minor effects on the expression profile of proteins in skin mucus and no effects on immune markers in splenocytes. BSFL meal showed no negative effect on liver and muscle health as indicated by plasma alanine aminotranseferase, asparate aminotransferase and creatine kinase. The present study showed that replacing conventional protein sources with low to moderate levels of BSFL meal (6.25% and 12.5%) or paste (3.7% and 6.7%) reduced enterocyte steatosis in pyloric caeca, while replacing up to 25% with BSFL meal or 6.7% with paste improved distal intestine histology. Further, dietary inclusion of BSFL meal and paste had minor effects on skin mucus proteome and immune response in Atlantic salmon.

## Introduction

Insects represent great potential as a sustainable alternative to conventional protein sources in aquafeeds (1–3). Black soldier fly larvae (BSFL) (*Hermetia illucens*) has attracted attention as one of the most promising insect species to be used in feeds (4). This is mainly due to its high nutritional value with 31-59% protein, 11–49% lipid (3, 5, 6) and its ability to valorize low-quality organic material (7) and ensure sustainable industrial-scale production (4).

The effect of BSFL as a replacer of conventional protein sources such as fishmeal and plant protein on growth performance has been studied in several aquaculture fish species, including salmonids. Previous studies reported that BSFL can partially replace dietary protein sources without adverse effects on salmonid growth performance (8–14). However, when introducing a novel protein source into fish feed, assessment of health effects beyond the nutritional value is important. BSFL are known to contain bioactive compounds such as chitin (15, 16) and antimicrobial peptides (AMP) (17, 18) which have antioxidant and immunostimulatory properties in fish (19–22). Furthermore, BSFL contain high amounts of medium-chain fatty acid, lauric acid (C12:0) (14, 23), which has antimicrobial effects against gram-positive bacteria (24, 25). Others have reported that dietary inclusion of BSFL meal increased the abundance of beneficial microorganisms that contribute to the health of the host such as lactic acid (26–28) and butyrate (27) producing bacteria in the gut of rainbow trout (*Oncorhynchus mykiss*). Several studies have evaluated the effect of BSFL on salmonid health. Dietary inclusion of defatted BSFL meal (60%) or partially defatted BSFL meal (15%) did not compromise gut health in pre-smolt (29) and post-smolt Atlantic salmon (*Salmo salar*) (30), respectively. Further, partially defatted BSFL meal in diets (20–40%) caused no adverse effects on the histology of liver, spleen and gut in rainbow trout (31). In addition, defatted BSFL meal did not cause negative effect on liver health in salmon as indicated by decreased or unaltered activities of plasma markers of liver damage such as alanine aminotransferase (ALT) and aspartate aminotransferase (AST) (9, 32), and unaffected expression of genes related to stress response in the liver (32). On the contrary, Cardinaletti, Randazzo (13) reported an up-regulation of a stress-related gene in the liver of rainbow trout fed diets containing 21% full-fat BSFL meal for 98 days, suggesting a physiological activation of stress/inflammation response.

Previous studies focused on the health effects of dietary inclusion of dried BSFL meal for salmonids, in particular dried defatted BSFL meal for salmon. The present study investigated the nutritional value and health effects of two differently processed BSFL types in diets for pre-smolt Atlantic salmon. The two types of BSFL were low-processed and included full-fat dried BSFL meal and undried BSFL paste preserved with formic acid. The results on digestibility and utilization of nutrients and growth performance were reported by Weththasinghe, Hansen (14). In brief, BSFL meal and paste could replace low to moderate levels of dietary protein without compromising growth performance in Atlantic salmon (14). In addition, the fish in this study were sampled to investigate the effects on gut health, plasma biochemical parameters and immune response in pre-smolt salmon fed graded levels of BSFL meal and paste. Furthermore, according to our knowledge, none of the previous studies focused on the effect of dietary insects on the protein expression in skin mucus and distal intestine (DI) of fish. Therefore, we also analyzed the protein expression in skin mucus and DI using mass spectrometry and indirect enzyme-linked immunosorbent assay (ELISA). To further investigate the effect of dietary BSFL meal and paste on the immune system of fish, the phagocytic activity of head kidney (HK) macrophages-like cells isolated from salmon fed BSFL meal and paste was investigated in an *in vitro* challenge study with *Piscirickettsia salmonis* (*P. salmonis*).

## Materials and Methods

### Experimental Diets and Fish Rearing

Full-fat BSFL meal and paste were produced at HiProMine S.A., Poznan, Poland. BSFL from a same batch were used to produce dried BSFL meal and undried BSFL paste preserved with formic acid (2.5%). Seven isonitrogenous, isolipidic and isoenergetic diets were prepared according to the nutrient requirements of pre-smolt Atlantic salmon (33). The experimental diets consisted of a control diet based on fishmeal, plant protein sources (i.e. water-extracted soy protein concentrate, corn gluten, faba bean) and fish oil (Control-1); three diets with increasing levels of BSFL meal, substituting 6.25% (6.25IM), 12.5% (12.5IM) and 25% (25IM) of the protein content of Control-1. In addition, two diets with increasing levels of BSFL paste, substituting 3.7% (3.7IP) and 6.7% (6.7IP) of the protein of Control-1 and an extra control with 0.88% of formic acid (Control-2) were evaluated. Considering BSFL paste was preserved with formic acid, the Control-2 diet was included as a control for BSFL paste diets. The ingredient and chemical composition of the experimental diets are shown in **Table 1**. Further details on BSFL meal and paste and chemical composition and production of diets were reported by Weththasinghe, Hansen (14).

**Table 1:** Ingredient and analyzed chemical composition of experimental diets^1^.

	Control-1	6.25IM	12.5IM	25IM	Control-2	3.7IP	6.7IP
***Ingredients (%)***							
Fishmeal	25	23.24	21.48	17.69	25	20.27	16.62
Soy protein concentrate	35.5	33.45	30.92	25.58	35.5	29.18	23.92
Corn gluten	4	3.72	3.44	2.59	4	3.24	2.66
Faba bean	1.85	1.72	1.59	1.03	1.85	1.5	1.23
BSFL meal	0	8.07	16.13	32.27	0	0	0
BSFL paste	0	0	0	0	0	19.8	35.12
Wheat flour	13.64	13.64	13.64	13.64	13.64	11.91	10.56
Wheat bran	4	2.47	1.28	0	3.12	2.16	0.98
Fish oil	15	12.68	10.51	6.19	15	11.06	8.13
Formic acid	0	0	0	0	0.88	0	0
Yttrium oxide	0.01	0.01	0.01	0.01	0.01	0.01	0.01
Vit/min premix	0.65	0.65	0.65	0.65	0.65	0.57	0.5
Methionine	0.2	0.2	0.2	0.2	0.2	0.17	0.15
Choline chloride	0.15	0.15	0.15	0.15	0.15	0.13	0.12
***Chemical composition (%, as is)***							
Dry matter	92.9	92.5	91.5	90.8	91.1	91.9	91.9
Crude protein	46.8	47.4	46.4	45.7	45.8	47.2	47.9
Crude lipid	14.6	15.5	17.2	15.9	16.2	13	13.3
Starch	12.3	12.4	11.5	11.5	11.6	12	12.8
Ash	5.52	5.88	6.17	6.83	5.3	5.67	6.08
Formic acid	0	0	0	0	0.72	0.58	1.1
Gross energy (MJ kg**^-1^**)	21.9	21.7	21.7	21.5	21.6	21.4	21.1

^1^Control-1: Control diet. 6.25IM, 12.5IM and 25IM: black soldier fly larvae (BSFL) meal substituted 6.25%, 12.5% and 25% of protein content of Control-1. Control-2: Control diet with 0.88% of formic acid. 3.7IP and 6.7IP: BSFL paste substituted 3.7% and 6.7% of protein content of Control-1.

The fish experiment was conducted at the Center for fish research, Norwegian University of Life Sciences (NMBU). The experimental procedures were performed in accordance with the national guidelines for the care and use of animals (The Norwegian Animal Welfare Act and the Norwegian Regulation on Animal Experimentation). The details of the fish experiment were reported by Weththasinghe, Hansen (14). In brief, a total of 1260 Atlantic salmon (Aqua Gen Atlantic QLT-innOva SHIELD) with 34 g of mean initial weight were distributed into 21 fiberglass tanks (300 L capacity) with 60 fish per tank. Three replicate tanks were randomly assigned to each of the seven experimental diets. Fish were fed *ad libitum* (i.e. 10% excess) with experimental diets over a period of seven weeks. The fish were kept under continuous light in recirculated freshwater and the average water temperature was 14.8 °C during the experimental period.

### Sample Collection

At the end of the feeding period, six fish from each tank were randomly sampled, anesthetized with tricaine methanesulfonate (MS-222) (80 mg L^−1^). The fish in all tanks were fed up to three to four hours before sampling. The skin mucus was immediately collected from the skin using sterile plastic spatulas, avoiding bleeding and fecal contamination. The collected mucus was immediately frozen in liquid nitrogen and stored at -80 °C until analysis. After mucus collection, fish were euthanized by a sharp blow to the head. Blood was collected from the caudal vein and centrifuged. Plasma samples were kept on dry ice until transferred to -20 °C and stored at -80 °C.

The DI was defined as a darker color, larger diameter section of the intestine where annular rings were visible (34). The DI was opened longitudinally and, the content was removed carefully. For histology, a piece of DI and a piece of pyloric caeca (PC) were fixed in 4% phosphate-buffered formalin for 24 h at room temperature before storage in 70% ethanol until further processing. For protein extraction, a piece of DI was rinsed in phosphate-buffered saline (PBS) and placed in cryotubes, frozen in liquid nitrogen and stored at −80 °C. For flow cytometry analysis, three fish per tank were sampled, HK and spleen were removed under aseptic conditions into tubes containing L-15 medium (Sigma-Aldrich) and used immediately for extraction of macrophages-like cells and splenocytes, respectively.

### Histology

Histological sections of PC and DI (18 samples per dietary group) were processed by Aquamedic and at the Veterinary Institute Laboratory in Oslo, Norway according to their respective standard operating procedures. Briefly, formalin-fixed tissue samples were dehydrated in ethanol, equilibrated in xylene and embedded in paraffin. Sections of 3 μm thickness from each intestinal segment were prepared and stained with hematoxylin and eosin. The sections of PC and DI were then examined blindly by light microscopy with a focus on the morphological changes observed in soybean meal-induced enteritis as previously described for Atlantic salmon DI mucosa. The criteria included shortening of mucosal fold height, increase in width and cellularity of the submucosa and lamina propria, and loss of enterocyte supranuclear vacuolization (35). Additionally, for the PC, changes in the vacuolization of the intestinal enterocytes were evaluated. The degree of change for the different morphological characteristics evaluated for the PC and DI, was graded using a scoring system with a scale of 0–4 where 0 represented normal; 1, mild changes; 2, moderate changes; 3, marked changes and 4, severe changes.

### Detection of Immunological Markers by Indirect ELISA

The number of DI and skin mucus samples used for the detection of immunological markers were nine per dietary group, except for DI IgM and IgD, where 18 per dietary group were included. Samples were thawed on ice and homogenized using beads and ice-cold lysis buffer (Tris 20 mM, NaCl 100 mM, Triton X-100 0.05%, EDTA 5 mM, and protease inhibitor cocktail 1x) in a bead mill homogenizer (Qiagen RETSCH tissuelyser). Then, the homogenate was centrifuged at 12000 x g for 25 min at 4 °C. The supernatant, containing soluble proteins, was then transferred to new tubes on ice and stored at -20 °C until use. All protein samples were quantified by a Pierce BCA Protein Assay Kit (Thermo Fisher Scientific) following the manufacturer’s instructions. The extracted soluble proteins from DI and skin mucus were used for the detection of IgM, IgD, IFNγ and IL-1β using indirect ELISA. Briefly, each sample was diluted in carbonate buffer (60 mM NaHCO3 pH 9.6) to 45 ng μL^−1^ and 100 μL of diluted samples were seeded (in duplicate) in a 96-well plate (Nunc) for overnight incubation at 4 °C. Next, 200 μL of blocking solution (5% Blotting-Grade Block (BioRad) diluted in PBS was added to each well and incubated for 2 h at 37 °C. Next, 50 μL of the primary antibody was added to each well and plates were incubated for 90 min at 37 °C. The primary antibodies used were as follows: monoclonal anti-IgD, monoclonal anti-IgM, polyclonal anti-IFNγ or polyclonal anti-IL-1β at 1:200 dilution as reported by Sahlmann, Djordjevic (36) and kindly donated by Dr. Luis Mercado. Next, 50 μL of a secondary antibody diluted to 1:7000 (goat anti-mouse IgG-HRP or mouse anti-rabbit IgG-HRP) was added and incubated for 60 min at 37°C. Finally, 100 μL of chromagen substrate 3,3′,5,5′-tetramethylbenzidine single solution (TMB, Thermofisher) was added and incubated for 30 min at room temperature. The reaction was stopped with 50 μL of 1 N sulfuric acid and read at 450 nm on a Spectra Max microplate reader (Spectra Max M2; Molecular Devices).

### Plasma Analysis for Biochemical and Immune Parameters

The ferric reducing ability of plasma (FRAP), ALT, AST, creatine kinase (CK), C-reactive protein (CRP) and lysozyme in plasma were analyzed (18 samples per dietary group) at Skretting Aquaculture Research Centre (ARC), Stavanger, Norway. The ALT, AST, CK and CRP of plasma samples were analyzed using kits from Thermo Fisher Scientific (article numbers: ALT 981361, AST 981363, CK 981828, CRP 981934) on a Konelab 30i Chemistry Analyzer (Thermo Fisher Scientific). Plasma FRAP and lysozyme content were analyzed using in-house protocols adapted from Benzie and Strain (37) and Parry, Chandan (38), respectively.

### Flow Cytometry and Phagocytic Capacity

HK and spleen (three fish per tank and pooled) leukocytes were isolated, according to Iliev, Thim (39). To investigate the surface expression of IgD, IgM and CD8 in splenocytes, the samples were washed with ice-cold PBS and incubated with primary antibody (anti-IgD at 1:400 dilution, anti-IgM at 1: 400 dilution or anti-CD8 at 1:200 dilution) for 1 h in PBS, 5% FBS on ice. Then the cells were incubated with secondary Alexa546 coagulated antibody diluted to 1 μg/ml in PBS, 5% FBS for 30 min on ice. Thereafter, cells were washed twice in PBS and analyzed by flow cytometry using a Beckman Coulter Gallios flow cytometer.

The capacity of HK cells to uptake *P. salmonis in vitro* was measured according to methods described by Lagos, Tandberg (40). Briefly, 1 ml of isolated adherent HK leukocytes (i.e. HK macrophages-like cells) (1 × 10^6^ cells/ml) per sample was incubated for 1 h at 15°C with inactivated CFSE labeled *P. salmonis* (1 × 10^7^ CFU/ml), or PBS as a control, without any centrifugation step to enhance the infection. After the incubation, cells were centrifuged at 600 × g for 10 min and the pellet was washed three times with ice-cold PBS and analyzed by flow cytometry using a Beckman Coulter Gallios flow cytometer. The fluorescence of CFSE-conjugated *P. salmonis* was measured before and after the addition of trypan blue (0.025% final concentration) to quench extracellular fluorescence. Data were analyzed using Kaluza software v.1.2 (Beckman Coulter) and at least 10,000 events were collected for each sample.

For morphological characterization, HK macrophages-like cells co-cultured with CFSE-conjugated *P. salmonis* were seeded in an 8-chamber tissue cultured treated glass Falcon CultureSlide® (Corning, New York, USA) at a density of 150,000 cells per chamber. After 1 h, cells were washed with PBS and fixed with 3% paraformaldehyde (Sigma-Aldrich) for 20 min at 4°C. Then, cells were washed three times for 3 min with PBS and left to air dry. Once dried, plastic chambers were removed from the slides. Three drops of mounting medium, Fluoroshield (Sigma-Aldrich), containing DAPI were added to the slides and covered with a coverslip. Confocal laser microscopy (Zeiss LSM 800) was used for imaging and the images were analyzed by ImageJ software.

### Skin Mucus Proteomics

The extracted and quantified soluble protein from skin mucus (three samples per dietary group) were used for mass spectrometry analysis. Briefly, 20 µg of total protein in PBS were pH adjusted to 8 by adding ammonium bicarbonate (Sigma-Aldrich, Darmstadt, Germany). The samples were then digested with 1 µg trypsin (Promega, sequencing grade) overnight at 37 °C. The tryptic peptides were analyzed using an Ultimate 3000 RSLCnano-UHPLC system connected to a Q Exactive liquid chromatography-mass spectrometer (LC-MS/MS) (Thermo Fisher Scientific, Bremen, Germany). LC-MS/MS was run at the Proteomics Core Facility (PCF) at the University of Oslo, Norway. The acquired raw data were analyzed using MaxQuant (41) version 1.4.1.2. and Perseus version 1.6.0.7 based on MS1 intensity quantification. Proteins were quantified using the MaxLFQ algorithm (42). The data were searched against the salmon proteome (82390 sequences, June 2019). Peptide identifications were filtered to achieve a protein false discovery rate (FDR) of 1% using the target-decoy strategy. The analysis was restricted to proteins identified in at least two of the three replicates per dietary group. Protein raw data were transferred to log normalization and then volcano plot analysis, multivariate statistical analysis and data modeling were performed in R (R Core Team, 2019) using the package DEP (43) and vsn (44). In addition to differentially expressed proteins, the proteins uniquely expressed in each diet were identified. UniprotKB database was used for the functional annotation of the proteins. The potential functions of these proteins were inferred from the homologs for their UniprotKB sequence. The mass spectrometry proteomics data have been deposited to the ProteomeXchange Consortium via the PRIDE (45) partner repository with the dataset identifier PXD019125.

### Statistical Analysis

Differences in histological scores for the morphological characteristics of the DI and PC tissue were analyzed for statistical significance using ordinal logistic regression run in the R statistical package (version 3.4.2; 2017) within the RStudio interphase (version 1.1.383; 2017). Three different group comparisons were conducted, and these comprised of: comparison 1, between Control-1, 6.25IM, 12.5IM and 25IM diets, comparison 2, between Control-1, Control-2, 3.7IP, and 6.7IP and comparison 3, between Control-2, 3.7IP, and 6.7IP. Differences were examined based on odds ratios of the different dietary groups having different histology scores compared to the reference diet. Reference diets were Control-1 for comparison 1 and 2 and Control-2 for comparison 3.

The biochemical and immune parameters of plasma, results of ELISA, surface expression of IgD, IgM and CD8 in splenocytes and HK macrophages-like cell phagocytic activity were analyzed using one-way ANOVA, followed by Tukey’s multiple comparison test for comparison of means. Differences at *p*<0.05 were considered as significant. In addition, polynomial contrast analysis was used to evaluate the relationship between plasma parameters and dietary BSFL meal or paste levels. The results of ELISA were presented as fold changes relative to Control-1 and two different group comparisons were conducted, and these comprised of: comparison 1, between 6.25IM, 12.5IM and 25IM diets and comparison 2, between Control-2, 3.7IP, and 6.7IP diets. The means of each diet were also compared with Control-1 diet using Dunnett’s multiple comparison test. The statistical analyses of plasma parameters were performed using IBM SPSS Statistics 26 software, whereas statistical analyses of ELISA results, surface expression of IgD, IgM and CD8 in splenocytes and HK macrophages-like cell phagocytic activity were performed using GraphPad Prism 8.3.1. Prior to ANOVA, these data were tested for homogeneity of variance by Levene’s test and normal distribution of residuals was checked using Kolmogorov-Smirnov test. These two tests were performed in IBM SPSS Statistics 26 software. When the assumption of equal variance was violated, the data were tested using Brown-Forsythe ANOVA test and Welch’s ANOVA test, followed by Dunnett’s T3 multiple comparison test. Kruskal-Wallis test was used when the data were not normally distributed or the data showed both heterogeneity of variances and non-normal distribution, and followed by Dunn’s multiple comparison test. These tests were performed in GraphPad Prism 8.3.1.

## Results

### Gut Health Assessment

#### Pyloric Caeca

**Figure 1:**
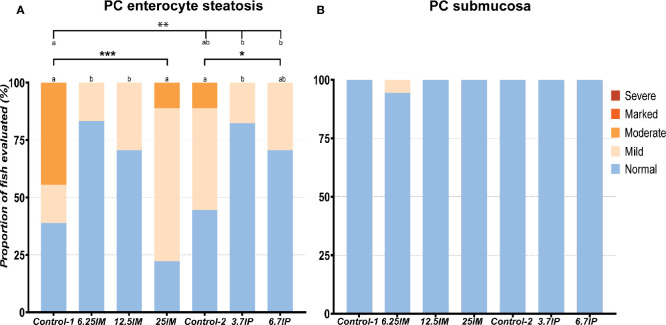
Histological evaluation of pyloric caeca (PC). **(A)** Number of PC tissue sections that were scored “normal”, “mild”, moderate”, “marked” or “severe” for enterocyte steatosis and **(B)** increase in the width and inflammatory cell infiltration in the submucosa. Column charts, within each of the demarcation bars that mark Comparison 1 (Control-1, 6.25IM, 12.5IM and 25IM), Comparison 2 (Control-1, Control-2, 3.7IP, and 6.7IP) and Comparison 3 (Control-2, 3.7IP and 6.7IP), that do not share superscript letters are statistically different (*p* < 0.05). Asterisks denote level of significance (*p < 0.05, **p < 0.01, ***p < 0.001) following outcomes of an ordinal logistic regression for differences in distribution of histological scores between the dietary groups. Each measurement was performed in triplicate using 6 fish per tank. Control-1: Control diet. 6.25IM, 12.5IM and 25IM: BSFL meal substituted 6.25%, 12.5% and 25% of protein content of Control-1. Control-2: Control diet with 0.88% of formic acid. 3.7IP and 6.7IP: BSFL paste substituted 3.7% and 6.7% of protein content of Control-1.

Mild to moderate accumulation of lipid in vacuoles in the epithelial cells, also called enterocyte steatosis was the main morphological change observed in the PC of all diet groups, as illustrated in **Supplementary Figure 1**. High occurrence and severity of the enterocyte steatosis was observed in Control-1, Control-2 and 25IM (**Figure 1A**). The degree of the steatosis was lower (less in number and severity of the steatosis) (*p*<0.01) in 6.25IM, 12.5IM, 3.7IP and 6.7IP diets fed fish compared to Control-1 (**Figure 1A**). The PC submucosa of fish fed all the diets were normal with regards to other morphological changes, i.e. increase in the width and inflammatory cell infiltration in the submucosa (**Figure 1B**).

#### Distal Intestine

**Figure 2:**
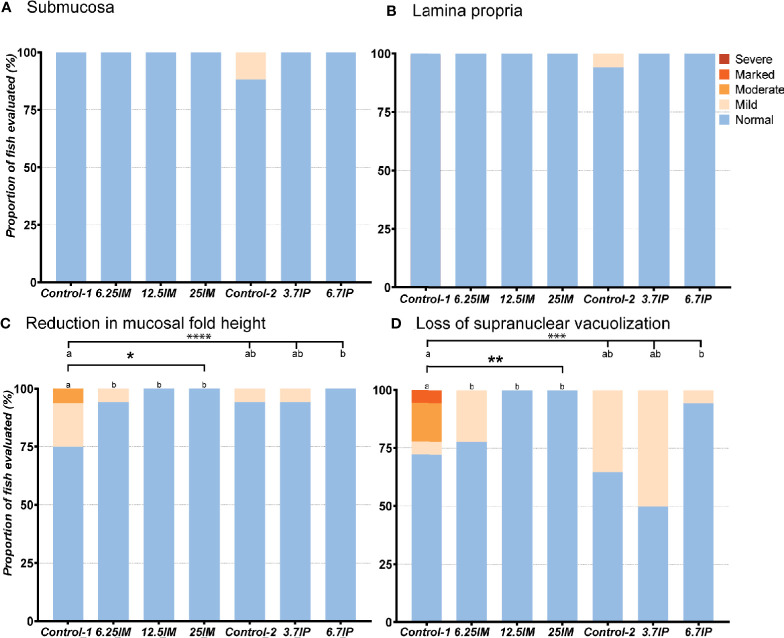
Histological evaluation of distal intestine (DI). **(A)** Number of DI tissue sections that were scored “normal”, “mild”, “moderate”, “marked or “severe” for the morphological characteristics of increase in width and inflammatory cell infiltration of the submucosa and **(B)** lamina propria, **(C)** reduction in mucosal fold height and **(D)** loss of enterocyte supranuclear vacuolization. Column charts, within each of the demarcation bars that mark Comparison 1 (Diets Control*-*1, 6.25IM, 12.5IM and 25IM) and Comparison 2 (Control*-*1, Control*-*2, 3.7IP, and 6.7IP), that do not share superscript letters are statistically different (p<0.05). Asterisks denote level of significance (* = p<0.05, ** = p<0.01, *** = p<0.001, **** = p<0.0001) following outcomes of an ordinal logistic regression for differences in distribution of histological scores between the dietary groups. Each measurement was performed in triplicate using 6 fish per tank. Control-1: Control diet. 6.25IM, 12.5IM and 25IM: BSFL meal substituted 6.25%, 12.5% and 25% of protein content of Control-1. Control-2: Control diet with 0.88% of formic acid. 3.7IP and 6.7IP: BSFL paste substituted 3.7% and 6.7% of protein content of Control-1.

Evaluation of the DI revealed normal and healthy morphology for most of the fish in the present study (**Figure 2**). Increased width and infiltration of the submucosa and lamina propria by inflammatory cells in DI were not observed in any of the groups except for focal lesions in two fish fed Control-2 diet (**Figures 2A, B**). The fish fed Control-1 showed mild inflammation changes (**Supplementary Figure 1**) characterized by a mild to moderate mucosal fold shortening (**Figure 2C**) due to a loss in enterocyte vacuolization (**Figure 2D**). Similar to Control-1, the Control-2 and 3.7IP diets also showed inflammatory changes in terms of mucosal fold shortening (**Figure 2C**) and loss of supranuclear vacuolization (**Figure 2D**). However, BSFL meal diets and 6.7IP diet fed fish showed no such inflammatory changes in the DI (**Figures 2C, D**).

### Immune Parameters in Distal Intestine and Skin Mucus

**Figure 3:**
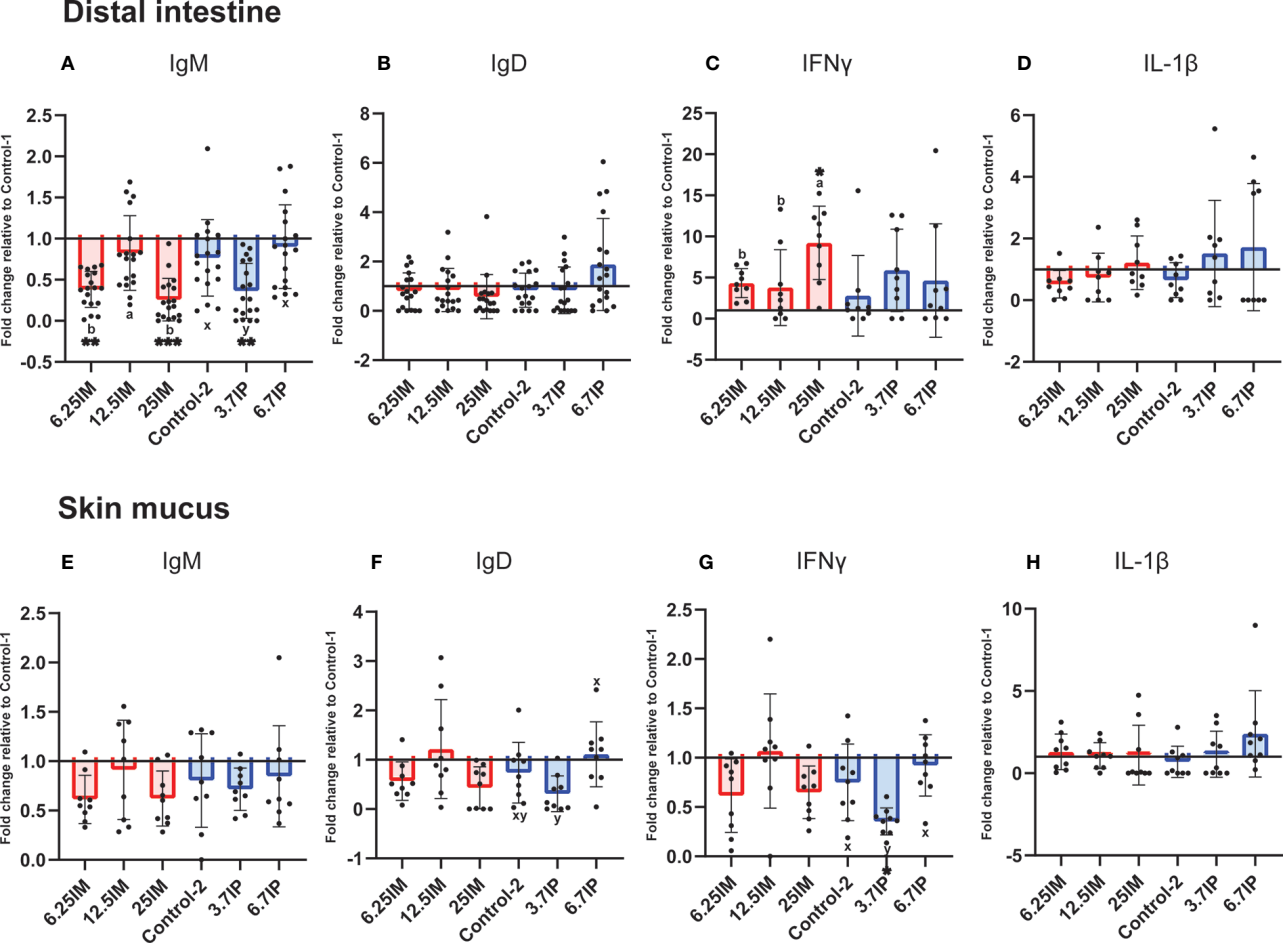
Detection of immunoglobulin and pro-inflammatory cytokine levels in distal intestine and skin mucus of fish fed experimental diets containing black soldier fly larvae (BSFL) meal and paste. **(A)** IgM, **(B)** IgD, **(C)** IFNγ and **(D)** IL-1β levels in distal intestine and **(E)** IgM, **(F)** IgD, **(G)** IFNγ and **(H)** IL-1β levels in skin mucus. Results are expressed as fold change relative to Control-1. Error bars indicate standard deviation. Error bars that are labelled with different superscript letters are significantly (p<0.05) different according to Tukey’s multiple comparison test or Dunn’s multiple comparison test. The letters a-b denote significant differences among 6.25IM, 12.5IM and 25IM diets (Comparison 1), whereas x–y denote significant differences among Control-2, 3.7IP and 6.7IP (Comparison 2). Asterisks denote level of significant difference with Control-1 (*= p<0.01, ** = p<0.001, *** = p<0.0001) according to Dunnett’s multiple comparison test or Dunn’s multiple comparison test. Control-1: Control diet. 6.25IM, 12.5IM and 25IM: BSFL meal substituted 6.25%, 12.5% and 25% of protein content of Control-1. Control-2: Control diet with 0.88% of formic acid. 3.7IP and 6.7IP: BSFL paste substituted 3.7% and 6.7% of protein content of Control-1.

Immunoglobulin and pro-inflammatory cytokine levels in the DI and skin mucus are shown in **Figure 3** as fold changes relative to Control-1. Considering BSFL paste was preserved in formic acid, we also compared the effect of BSFL paste diets relative to Control-2 (**Supplementary Figure 2**).

IgM level was lower (*p*<0.001) in DI of fish fed 6.25IM (0.4-fold change) and 25IM (0.3-fold change) diets, relative to Control-1, while fish fed 12.5IM diet showed higher IgM level (*p*<0.01) compared to 6.25IM and 25IM diets (**Figure 3A**). The 25IM diet showed a 9.2-fold increase (*p*<0.01) of DI IFNγ relative to Control-1. DI IFNγ in 25IM was also higher (*p*<0.05) than other BSFL meal diets (**Figure 3C**). The IgD and IL-1β levels in the DI were not affected by the dietary inclusion of BSFL meal (**Figures 3B, D**). In BSFL paste fed salmon, the 3.7IP diet showed a 0.4-fold decrease (*p*<0.001) in DI IgM relative to Control-1 (**Figure 3A**). The DI IgM level in the 3.7IP diet was also lower (*p*<0.05) than Control-2 and 6.7IP. The IgD, IFNγ and IL-1β levels in the DI were not affected by the dietary inclusion of BSFL paste (**Figures 3B–D**).

In the skin mucus, the IgM, IgD, IFNγ and IL-1β levels were not affected by the dietary inclusion of BSFL meal (**Figures 3E–H**). Similarly, BSFL paste did not change the skin mucus IgM and IL-1β levels (**Figures 3E, H**). However, the fish fed 3.7IP diet showed a lower (*p*<0.05) IgD level than 6.7IP diet (**Figure 3F**). Further, 3.7IP diet also showed a 0.4-fold decrease (*p*<0.01) of IFNγ level relative to Control-1, which was also lower (*p*<0.05) than Control-2 and 6.7IP diets (**Figure 3G**). We observed high variations in immunoglobulin and cytokine levels in the DI and skin mucus among the fish within groups.

### Plasma Immune and Biochemical Parameters

Plasma FRAP, ALT, AST, CK, CRP and lysozyme contents in BSFL meal and paste diets were not statistically different from Control-1 (**Table 2**). Plasma lysozyme content was higher (*p* <0.01) in 12.5IM fed fish compared to 6.25IM fed fish. Plasma FRAP content was higher (*p* <0.01) in 6.7IP compared to Control-2. According to polynomial contrast analysis, there was a negative linear relationship between plasma CRP and dietary BSFL meal level (*p* <0.05). Also, the plasma FRAP content increased linearly with increasing dietary BSFL paste level (*p* <0.01)). In addition, there was a quadratic relationship between plasma AST and dietary BSFL paste level (*p* <0.05) with the highest level at 3.7%, and the same trend was observed between plasma CK and dietary BSFL paste level (*p* = 0.07).

**Table 2:** Immune and biochemical parameters in plasma of fish fed black soldier fly larvae (BSFL) meal and paste^1^.

									*Comparison 1*—*BSFL meal diets^2^*			*Comparison 2*—*BSFL paste diets^2^*		
	Control-1	6.25IM	12.5IM	25IM	Control-2	3.7IP	6.7IP	SEM^3^	*p_value_* ^4^	*p*_linear_^4^	*p*_quad_^4^	*p_value_* ^5^	*p*_linear_^5^	*p*_quad_^5^
Ferric reducing antioxidant power (FRAP) (μmol/L)	920.5^XY^	961.4	961.4	979.1	889.9^Y^	993.0^XY^	1017.5^X^	11.55	0.52	0.19	0.58	0.005	0.005	0.40
Alanine aminotransferase (ALT) (U/L)	14.3	13.1	18.2	14.8	14.2	13.7	11.8	0.78	0.09	0.68	0.40	0.55	0.35	0.69
Aspartate aminotransferase (AST) (U/L)	637.4	538.2	652.7	532.9	530.3	685.5	535.9	18.72	0.15	0.27	0.67	0.09	0.82	0.016
Creatine kinase activity (CK) (U/L)	19739.4	16880.3	23553.1	18441.1	17438.0	25792.3	21333.8	997.7	0.09	0.98	0.41	0.13	0.27	0.07
C-reactive protein (CRP) (mg/L)	2.7	2.3	1.1	0.5	0.8	1.6	0.9	0.23	0.07	0.018	0.75	0.04	0.67	0.11
Lysozyme (U/ml)	1199.9^ab^	1040.5^b^	1339.1^a^	1198.3^ab^	1236.9	1185.9	1266.5	20.84	0.003	0.35	0.47	0.63	0.73	0.29

^1^Control-1: Control diet. 6.25IM, 12.5IM and 25IM: BSFL meal substituted 6.25%, 12.5% and 25% of protein content of Control-1. Control-2: Control diet with 0.88% of formic acid. 3.7IP and 6.7IP: BSFL paste substituted 3.7% and 6.7% of protein content of Control-1.

^2^Two group comparisons were conducted: Comparison 1, between Control-1, 6.25IM, 12.5IM and 25IM diets; Comparison 2, between Control-1, Control-2, 3.7IP, and 6.7IP.

^3^Standard error mean.

^4^p values for comparison 1.

^5^p values for Comparison 2.

p_value_: p value for one-way ANOVA, Kruskal-Wallis test or Welch’s ANOVA test. Different superscript letters of lysozyme values of Control-1, 6.25IM, 12.5IM and 25IM diets (Comparison 1) indicate significant (p<0.05) differences according to Tukey’s multiple comparison test. Different superscript letters of FRAP values of Control-1, Control-2, 3.7IP and 6.7_IP diets (Comparison 2) indicate significant (p<0.05) differences according to Dunnett’s T3 multiple comparison test. p_linear_ and p_quad_ are the p values of linear and quadratic components of the polynomial contrast analysis between each plasma parameter and BSFL meal/paste protein level in the diet: Control-1 was excluded in the polynomial contrast analysis of BSFL paste diets (Comparison 2).

### Immune Markers in Spleen and Phagocytic Activity in Head Kidney Macrophages-Like Cells

**Figure 4:**
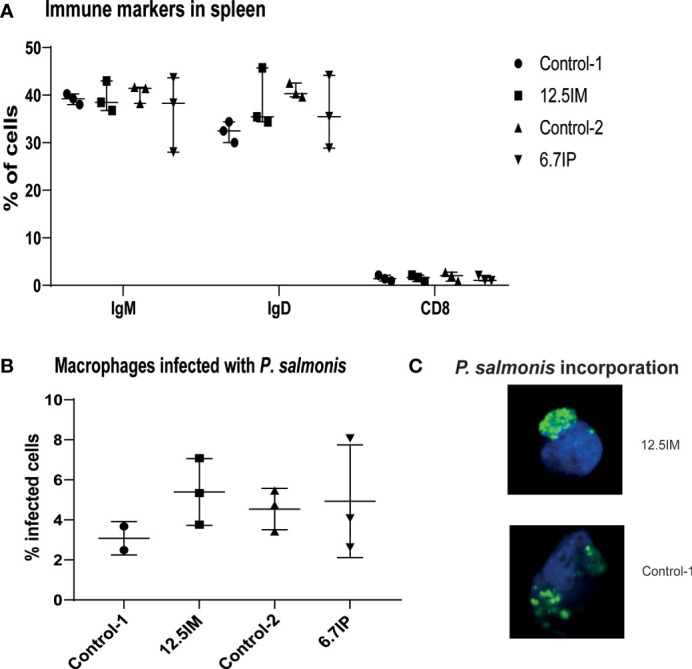
Expression of immune markers of splenocytes and phagocytic capacity of head kidney macrophages-like cells isolated from salmon fed experimental diets containing black soldier fly larvae (BSFL) meal and paste. **(A)** Flow cytometry showing the number of splenocytes expressing IgM, IgD or CD8. **(B)** number of macrophages-like cells able to incorporate labeled-bacteria (*P. salmonis* CFSE labelled). **(C)** confocal microscopy confirming the incorporation of labelled *P. salmonis*. Each measurement was performed using 3 fish per tank. Control-1: Control diet; 12.5IM: BSFL meal substituted 12.5% of protein content of Control-1; Control-2: Control diet with 0.88% of formic acid; 6.7IP: BSFL paste substituted 6.7% of protein content of Control-1.

Considering the results presented above, the immune markers in spleen and phagocytic activity in HK macrophages-like cells were measured only in four diets, i.e. Control-1, 12.5IM, Control-2 and 6.7IP. The number of IgM+, IgD+ and CD8+ splenocytes was not affected by dietary treatments (**Figure 4A**). Further, dietary treatments did not affect the phagocytic activity in HK macrophages-like cells. However, macrophages-like cells isolated from HK of fish fed 12.5IM diet were more prone to incorporate labeled-*P. salmonis* compared to other diets, although it was not statistically significant (**Figure 4B**). The incorporation of *P. salmonis* was confirmed by confocal microscopy (**Figure 4C**).

### Skin Mucus Proteomics

The proteins present in the skin mucus of fish fed BSFL meal or paste were analyzed by mass spectrometry. A total of 1636 salmon proteins were identified (**Supplementary Table 1**). After filtering for proteins presented in at least two of the three replicates per dietary group, 968 proteins were selected for further analyses. Volcano plots displaying normalized log2 of LFQ (label-free quantification) protein abundance ratio between Control-1 and other experimental diets, or between Control-2 and BSFL paste diets, against statistical significance measurements (−log10 *p* value) are shown in **Supplementary Figure 3**. The dietary inclusion of BSFL meal and paste had minor effects on the expression profile of proteins. The exception was the expression of apolipoprotein D and NAD(P)H dehydrogenase [quinone] 1- like in 25IM diet compared to Control-1. In addition, 12.5IM diet over-expressed heterogeneous nuclear ribonucleoprotein A0-like and reduced the expression of RNA-binding protein cabeza-like and serum deprivation-response protein compared to Control-1. Further, compared to Control-2, the 3.7IP diet showed lower expression of beta-globin (**Table 3**).

**Table 3:** Differentially expressed proteins in the skin mucus of fish fed black soldier fly larvae (BSFL) meal or paste^1^.

Diet	SwissProt accession no	Protein name	Gene name	Fold change	Adjusted *p* value
**Abundance compared to Control-1**					
**12.5IM**	A0A1S3RTG2	Heterogeneous nuclear ribonucleoprotein A0-like	*LOC106604941*	2.27	0.024
**12.5IM**	A0A1S3S0N2	RNA-binding protein cabeza-like	*LOC106606097*	−2.65	0.024
**12.5IM**	A0A1S3PL20	Serum deprivation-response protein	*sdpr*	−0.64	0.024
**25IM**	B5XEY8	Apolipoprotein D	*APOD*	1.58	0.003
**25IM**	A0A1S3PWS4	NAD(P)H dehydrogenase [quinone] 1-like	*LOC106587985*	−1.61	0.004
**Abundance compared to Control-2**					
**3.7IP**	Q91470	Beta-globin	*HBB1*	−3.53	0.028

^1^Control-1: Control diet. 12.5IM and 25IM: BSFL meal substituted 12.5% and 25% of protein content of Control-1. Control-2: Control diet with 0.88% of formic acid. 3.7IP: BSFL paste substituted 3.7% of protein content of Control-1.

The detected salmon proteins were checked for unique proteins. The criteria used for the identification of unique proteins was the presence of the particular protein in at least two of the three replicates in a dietary group. The unique proteins expressed in the skin mucus of fish fed experimental diets are shown in **Supplementary Table 2**. The BSFL meal diets and 6.7IP fed fish uniquely expressed several immune-related proteins, whereas 25IM also uniquely expressed superoxide dismutase.

## Discussion

The present study reports the effect of dietary inclusion of graded levels of BSFL meal and paste on gut health, plasma biochemical parameters, immune response and protein expression in skin mucus in pre-smolt salmon.

The gut is considered as the main site of exposure to nutrients and antigens (46). In a normal and healthy gut, almost no vacuolization is observed in the enterocytes of the proximal intestine (30). Increased vacuolization of the enterocytes in the PC is called enterocyte steatosis, which reflects an abnormal lipid droplet accumulation within the enterocytes due to impaired lipoprotein synthesis (47, 48) or lipid transport across the intestinal mucosa to the circulatory system (49). This condition is observed in fish affected by the so-called lipid malabsorption syndrome (50). The extensive accumulation of lipid droplets in enterocytes might cause damage to the epithelium affecting the integrity of the epithelial barrier. Such damage can translocate pathogenic or potentially pathogenic bacteria into the host and, having detrimental effects on fish health (47, 48). The observed mild to moderate enterocyte steatosis in the PC of fish fed the control diets in the present study might be due to the inclusion of high levels of plant ingredients (49–51) or lack of dietary choline (50, 52) as observed previously. However, a reduced enterocyte steatosis was observed in the PC of fish fed diets with low to moderate dietary BSFL meal and paste inclusion. Li, Kortner (30) and Li, Kortner (29) have also shown that post-smolt salmon fed diets containing 15% BSFL meal and pre-smolt salmon fed diets with 60% of de-chitinized BSFL meal presented a lower degree of enterocyte steatosis in the proximal intestine. The reason for the reduction in enterocyte steatosis in the BSFL meal and paste fed fish in the present study could be related to lower levels of plant ingredients or the presence of bioactive components in the BSFL such as choline. As reported by others, insects, including BSFL, are rich sources of choline (15, 53). Choline is important in lipid transport across the intestinal mucosa of salmon (50) and dietary choline chloride (0.37–0.4%) prevented excessive lipid accumulation in the proximal intestine in post-smolt Atlantic salmon (50, 52). In addition, the majority of fatty acids in BSFL meal were saturated (14) which might lead to reduced enterocyte steatosis, as observed in Arctic char (*Salvelinus alpinus*) by Olsen, Myklebust (48). On the contrary, we observed that 25% replacement of protein with BSFL meal caused mild-moderate enterocyte steatosis, similar to the control diets. This finding was associated with a lower lipid digestibility in fish fed diets with 25% replacement of protein with BSFL meal (14), suggesting that high level of chitin from BSFL could also be a causative factor for the enterocyte steatosis. The BSFL meal used in the present study contained 8% of chitin in dry matter basis, this corresponds to chitin levels of 0.6,1.2 and 2.3% for the meal diets and 0.4 and 0.6% for the paste diets (14). The diet that replaced 25% of protein with BSFL meal also had the highest level of lauric acid and this could also affect this condition. Further studies are thus needed to investigate the effect of lauric acid on the enterocyte steatosis in Atlantic salmon when fed BSFL.

Mild inflammatory changes were observed in the DI of fish fed the two control and 3.7IP diets in the present study. These inflammatory changes comprised predominantly of the shortening of the mucosal fold height due to the loss in the enterocyte supranuclear vacuolization. Loss of the vacuolization is also known to indicate a block in the enterocytic activity in the DI (54). As for enterocyte steatosis, the high inclusion of plant ingredients might lead to these changes in the present study (55, 56). Further, the soy protein concentrate that we used was water-extracted thus antinutritional factors such as saponin were not fully removed. Soya saponin is known to induce enteritis in the DI of Atlantic salmon (57–59). In accordance with the present results of BSFL meal diets and 6.7IP diet, others also reported that dietary inclusion of BSFL meal showed normal and healthy histology in DI of pre-smolt salmon (29) and rainbow trout (60) and mid intestine of post-smolt salmon (10). The reason for the absence of DI inflammatory changes in the BSFL meal diets and 6.7IP diet fed fish in the present study could be related to inclusion of BSFL or lower levels of plant protein ingredients. The fatty acid composition of BSFL might contribute for the absence of intestine inflammation. BSFL contain high amounts of medium-chain lauric acid (C12:0) (14, 23), which has antimicrobial effects against gram-positive bacteria (24, 25, 61, 62) and viruses (61, 62). Medium-chain fatty acids and medium-chain triglycerides have also been suggested to improve gut health under inflammatory conditions (63), which might be associated with the induction of the expression of host defense peptides in the gut (64). Another possible explanation for the absence of intestine inflammation in fish fed BSFL meal and paste may be related to BSFLs’ ability to modulate gut microbiota and increased microbial lactic acid and butyrate production. Although our study did not include microbiota analysis, others have reported that dietary inclusion of BSFL meal increased the abundance of lactic acid (26–28) and butyrate (27) producing bacteria in the gut of rainbow trout. It has been reported that lactate and butyrate could repair or prevent the intestinal damage caused by dietary soybean meal or oxidized soybean oil in fish (65, 66). The anti-inflammatory properties of microbe-derived butyrate in gut and its role in enhancing intestinal barrier function and mucosal immunity are well studied in human (67). It is also possible that the short-chain fatty acids including butyrate produced by gut microbiota might induce the expression of host defense peptides and prevent inflammation in the gut as observed in mammals and birds (64). Further experiments are needed to unravel the effect of the full-fat BSFL meal and paste on gut microbiota diversity of salmon.

Pro-inflammatory cytokines are known to be released as part of the innate immune response in fish (68). The dietary replacement of fishmeal and plant protein sources up to 12.5% with BSFL meal and 6.7% with BSFL paste, did not affect IL-1β or IFNγ levels in DI. This explains the absence of inflammatory changes in DI histology of fish fed these diets. Dietary inclusion of BSFL meal has been reported not to affect the intestinal expression of pro-inflammatory cytokine genes including IFNγ and IL-1β in pre-smolt (29) and post-smolt (30) salmon. Similarly, dietary inclusion of defatted BSFL meal did not affect the intestinal inflammatory cytokines concentrations of TNF-α, IL-6 and IL-8 in juvenile Japanese seabass (*Lateolabrax japonicus*) (69). In contrast, high (25IM) inclusion level of BSFL meal induced high levels of IFNγ in DI. Thus, despite the absence of histological alterations in DI, a potential morphological effect might be expected in prolonged feeding with BSFL meal diets. Natural antibodies are crucial components of the innate humoral immune system, as they provide immediate, early and broad protection against pathogens (68). The IgD level in DI was not altered by the inclusion of BSFL meal and paste. However, the DI IgM presented low levels in 6.25IM, 25IM and 3.7IP diets fed fish compared to Control-1. The lower DI IgM might also be related to the absence of inflammatory changes in DI histology in BSFL meal containing diets, because elevated IgM level in intestinal mucosa might be a sign of inflammation (70). However, this effect was not observed in 3.7IP diet.

Oxidative stress within cells or tissue has adverse effects on fish health, thus antioxidants can have significant health-benefits (71). In the present study, the antioxidant capacity in the plasma was measured in terms of FRAP. The results of FRAP in the present study showed an increased plasma antioxidant capacity when increasing the level of dietary BSFL paste, whereas salmon fed BSFL meal showed unaltered plasma antioxidant defense capacity. Previous studies also reported that the activity of serum antioxidant enzymes did not alter or even increased with dietary inclusion of BSFL meal or pulp in Jian carp (*Cyprinus carpio* var. Jian) (72), yellow catfish (*Pelteobagrus fulvidraco*) (73) and mirror carp (*Cyprinus carpio* var. specularis) (74). However, Zhou, Liu (75) observed that serum antioxidant capacity was not affected by partial replacement of dietary fishmeal with BSFL meal in Jian carp, while complete replacement reduced serum antioxidant capacity.

ALT and AST are enzymes present in liver and spleen, and leak into the bloodstream upon liver cell damage; therefore, high levels of these enzymes in blood are indicators of the liver damage (76). On the other hand, CK is concentrated in muscle and heart tissue and CK in the blood indicates damage of these tissues (77). In the present study, plasma AST, ALT and CK levels were not affected by BSFL meal, suggesting that dietary BSFL meal might not affect liver and muscle health. In accordance with the present study, several studies demonstrated that the activities of serum ALT and AST were not altered or in some cases even decreased by dietary inclusion of BSFL meal or pulp in pre-smolt (32) and post-smolt salmon (9), Jian carp (72), Japanese seabass (69) and mirror carp (74). In addition, Belghit, Waagbø (32) and Vargas-Abúndez, Randazzo (78) observed that dietary inclusion of BSFL meal did not affect the expression of genes involved in stress response (heat‐shock protein‐70 and superoxide dismutase) in the liver of pre-smolt salmon and clownfish (*Amphiprion ocellaris*) respectively, suggesting no induction of stress response and further confirming that BSFL ingredients did not cause any negative effect on liver health. On the contrary, increased expression of heat‐shock protein‐70 gene was observed in the hepatopancreas of Jian carp fed BSFL meal diets, suggesting an induced stress response only when dietary substitution of fishmeal exceeded 75% (72). In agreement with the present results, dietary inclusion of BSFL meal did not affect the plasma CK level in rainbow trout (60) or intestinal CK in Japanese seabass (69). These results might be associated with the diminished CRP level in the plasma of fish fed BSFL meal. CRP is an acute-phase serum protein and a mediator of innate immunity (79). The blood CRP level is increased in response to acute infection, inflammation or tissue injury (80, 81). Further, the serum CRP level was increased in rainbow trout reared in unfavorable environment, i.e. high-water temperature (82). The diminished CRP level in the plasma of fish fed BSFL meal in the present study might also be associated with the absence of DI inflammatory changes in BSFL meal diets. However, according to the polynomial contrast analysis, plasma AST and CK levels tend to increase at 3.7% replacement of protein with BSFL paste.

Lysozyme is an important defense molecule of the innate immune system of fish (83, 84), which is important in mediating protection against microbial invasion. Lysozyme is distributed in mucus, lymphoid tissue, plasma and other body fluids of freshwater and marine fish (84). It has been reported that dietary defatted BSFL meal did not affect the serum lysozyme activity in Japanese seabass (69). However, we observed low lysozyme level in fish fed 6.25IM, but increased level in fish fed 12.5IM. A dose-response of BSFL meal on serum lysozyme activity was observed in yellow catfish, with higher activity at lower dietary levels (73). Like B cells, macrophages are considered the principal phagocytic cell population in fish (85, 86) and phagocytosis is one of the main effector mechanisms of innate immunity against pathogens in fish (87). The HK macrophages-like cells of fish fed 12.5IM diet showed a numerically higher phagocytic activity when challenged by *P. salmonis*, the pathogen that cause Piscirickettsiosis in salmonid fish (88). A previous study also reported increased phagocytic activity of peritoneal leukocytes in red sea bream (*Pagrus major*) fed housefly pupae homogenate (89). Xiao, Jin (73) reported that dietary inclusion of BSFL meal did not affect the phagocytic index (Intracellular total bacterial count/Number of cells involved in phagocytosis) of white blood cells in yellow catfish, whereas the percentage of phagocytic cells involved in phagocytosis was lower at high dietary levels of BSFL meal (46–59%).

The skin mucus contains different innate immune parameters such as complements, lysozyme, immunoglobulins, cytokines, protease and lectins that protect fish against pathogens (90). As indicated by Esteban (90), the levels of immunoglobulin and pro-inflammatory cytokines in skin mucus varied between individuals and were detected in small quantities in the present study. The dietary inclusion of BSFL meal and paste did not affect the immunoglobulin and pro-inflammatory cytokine levels in the skin mucus, except the 3.7IP diet reduced both IgD and IFNγ in skin mucus. In accordance, mass spectrometry results also showed that the dietary inclusion of BSFL meal and paste had minor effects on the expression profile of proteins in the skin mucus. The higher inclusion of BSFL meal, replacing 25% of dietary protein, increased expression of apolipoprotein D which is involved in lipid transport. It has been demonstrated that apolipoprotein D gene expression *in vitro* was associated with several pathological and stressful conditions and pro-inflammatory stimuli in human cell lines (91). Further, 25IM diet also reduced the expression of NAD(P)H dehydrogenase [quinone] 1-like. NAD(P)H dehydrogenase [quinone] 1 isoform 1 gene is a biomarker of hepatotoxicity (92). The replacement of 3.7% of dietary protein with BSFL paste reduced the expression of beta-globin compared to formic acid containing control, which is a part of the hemoglobin complex and involved in oxygen transport.

The two diets with low and moderate levels of BSFL meal, i.e. 6.25IM and 12.5IM, uniquely expressed calreticulin-like. In a recent study, dietary inclusion of yeast cell wall extract increased abundance and expression of a calreticulin-like protein in the skin mucus of salmon (93). Further, calreticulin was over-expressed in the proteome of the DI of Atlantic salmon fed a probiotic feed additive 24 h after inducing inflammation. This suggest that it has a key role in many cellular and immunoregulatory functions, which help to counteract the inflammation (94). In addition, the fish fed 12.5IM diet uniquely expressed calpain-9-like and calpain-2 catalytic subunit-like. Calpains are calcium-dependent proteases (95, 96), which regulate phagocytosis and bacterial killing in macrophages (97). Further, the fish fed 12.5IM diet uniquely expressed high mobility group protein B1, H1 histone family member 0 like protein and galectin. High mobility group box 1 protein is known as an extra-cellular cytokine that triggers inflammatory and immune responses (98). Zhao, Hu (98) demonstrated that a high mobility group box 1 homolog from red drum (*Sciaenops ocellatus*) could function as a secreted cytokine in response to bacterial infection and promote innate defense through the activation of macrophages, and Xie, Hodgkinson (99) reported that in goldfish (*Carassius auratus*), high mobility group box 1 is a critical regulatory cytokine of inflammatory and antimicrobial response. Histone fragments or histone derived peptides from skin mucus of rainbow trout (100) and Atlantic salmon (101) were reported to possess antimicrobial properties. The 6.7IM diet fed fish uniquely expressed C-type lectin lectoxin-Thr1-like, which is a C-type lectin superfamily member and calpain small subunit 1. Fish lectins are reported to possess antimicrobial effects. In the presence of Ca^2+^, C-type lectins initiate a broad range of biological processes such as adhesion, endocytosis, and pathogen neutralization (102). The skin mucus of fish fed 25IM diet uniquely expressed superoxide dismutase, which is an antioxidant enzyme (103, 104) and a marker of stress response (32).

The minor effects of BSFL meal and paste on the immune response and skin mucus proteome in the present study indicate that the effect of BSFL might be more local, as observed in the gut, than systemic. In addition, many other reasons can explain the minor effects of BSFL on skin mucus proteome, such as the sampling time, i.e. seven weeks after feeding or the sampling method, i.e. scraping with plastic spatulas, which could influence the type and amount of protein obtained as shown in Fæste, Tartor (105). Besides, the fish were in the freshwater phase, which has been shown to have lower mucus viscosity than in seawater, meaning a different protein and glycosylation pattern (106). It is important to note that in the present study, trypsinized peptides were used in the mass spectrometry analysis, that might affect the protein conformation (107). It is possible that BSFL might have an effect on the conformation or glycosylation of skin mucus protein, which was not assessed in the present study.

In summary, the present study showed that 6.25IM diet reduced enterocyte steatosis in PC, improved DI histology, and reduced IgM level in DI. The fish fed 12.5IM diet reduced enterocyte steatosis in PC, improved DI histology, had a higher plasma lysozyme activity compared to 6.25IM, and tend to increase phagocytic activity in HK macrophages-like cells against *P. salmonis*. In addition, this diet showed uniquely expressed skin mucus proteins that regulate phagocytosis and antimicrobial responses, i.e. calpains and histone. On the other hand, 25IM diet improved DI histology, but showed enterocyte steatosis in PC, increased pro-inflammatory cytokine IFNγ in DI and reduced IgM in DI. Concurrently, this diet differentially expressed stress related proteins, i.e. over-expressed apolipoprotein D, reduced expression of NAD(P)H dehydrogenase [quinone] 1-like and uniquely expressed superoxide dismutase. In the case of BSFL paste diets, 3.7IP diet caused mild inflammatory changes in DI, reduced DI IgM and skin mucus IgD and IFNγ, and tend to increase plasma AST and CK, although it reduced enterocyte steatosis in PC. The 6.7IP diet reduced enterocyte steatosis in PC and improved DI histology, accompanied by higher plasma FRAP. Further, this diet uniquely expressed proteins that regulate phagocytosis and antimicrobial responses such as calpain and lectin. These results suggest that 6.25% and 12.5% replacement of dietary protein with BSFL meal and 6.7% replacement with BSFL paste, were more prone to cause positive impacts on gut health and immune response in Atlantic salmon, in comparison to low (3.7IP) and high inclusion levels (25IM). Further, the presence of formic acid in diets seemed to have no or minor effects on the gut, skin mucus and other general health parameters in plasma, spleen and HK. However, further studies with a longer feeding period or exposing fish to pathogenic challenges, are needed to confirm the significance of these results. The results observed in the present study might be attributed to the presence of chitin in the BSFL containing diets: 0.6% (6.25IM);1.2% (12.5IM); 2.3% (25IM) in BSFL meal diets and 0.4% (3.7IP); 0.6% (6.7IP) in BSFL paste diets (14). Chitin has complex and size-dependent effects on innate and adaptive immune responses (108), as chitin act as pathogen-associated molecular patterns (109). Large chitin polymers are biologically inert, while smaller fragments are pro-inflammatory, and even smaller fragments stimulate the production of anti-inflammatory cytokine (108). Da Silva, Hartl (109) reported that 40–70 µm sized chitin fragments could trigger inflammation and cytokine production via the pattern recognition receptors in mice. Numerous studies regarding the effect of chitin on the fish immune system indicated that chitin could be used as an immunostimulant when supplemented in fish diets (1, 21). However, chitin’s immunomodulating effect in fish has also been suggested to be dependent on the dietary inclusion level (13, 21). For instance, it has been reported that dietary inclusion of 1% chitin increased serum lysozyme activity in common carp (22), whereas <1% inclusion did not alter serum lysozyme activity and phagocytic activity of HK leukocytes in gilthead seabream (*Sparus aurata*) (19). However, Esteban, Cuesta (19) also reported that administration of a chitin diet (<1%) enhanced seabream immune activity through the non-specific modulation of haemolytic complement activity, leucocyte respiratory burst activity and cytotoxicity in a dose-dependent and a time-dependent manner. As discussed by Sánchez-Muros, Barroso (110) dietary incorporation of chitin stimulated macrophage activity in rainbow trout. Chitin or chitosan enriched diet (1%) could also modulate the immune system and the disease resistance in *Cirrhina mrigala* (111). Chitin and its derivatives such as chitosan, were also reported to have antioxidant properties (112–114). In addition to chitin, BSFL also contain AMP (17, 18) which could also contribute for the present results. A previous study also reported that dietary inclusion of AMP could improve immunity and oxidation resistance in common carp (*Cyprinus carpio*) (20). It seems that the stimulation of the immune system has a small window of activation that might be triggered by the concentration of chitin, AMP or other components present in the BSFL containing diet. Hence, future studies should investigate the effect of graded levels of various bioactive components of BSFL on gut health and immune response in fish.

## Conclusion

The present study showed that replacing conventional protein sources with low to moderate levels of BSFL meal (6.25% and 12.5%) or paste (3.7% and 6.7%) reduced enterocyte steatosis in pyloric caeca, while replacing up to 25% with BSFL meal or 6.7% with paste improved distal intestine histology. The increasing BSFL meal level in the diet linearly decreased plasma C-reactive protein, while increasing BSFL paste linearly increased plasma antioxidant capacity. The dietary inclusion of BSFL meal and paste had minor effects on skin mucus proteome and immune response in Atlantic salmon.

## Data Availability Statement

The datasets presented in this study can be found in online repositories. The names of the repository/repositories and accession number(s) can be found below: https://www.ebi.ac.uk/pride/archive/, PXD019125.

## Ethics Statement

Ethical review and approval was not required for the animal study because the fish experiment was conducted at Center for Fish Research at Norwegian University of Life Sciences (NMBU), which is a research facility approved by Norwegian Animal Research Authority (permit no. 109) and operates in accordance with Norwegian Regulations of 17th of June 2008 No. 822: Regulations relating to Operation of Aquaculture Establishments (Aquaculture Operation Regulations). The experimental procedures were performed in accordance with the national guidelines for the care and use of animals (The Norwegian Animal Welfare Act and the Norwegian Regulation on Animal Experimentation). All the experimental diets were formulated to meet the known nutrient requirement of salmon (NRC, 2011); thus, the fish were not exposed to nutrient deficiencies during the experiment. Insects are found in aquatic environments and part of the natural diets of salmon. The other ingredients used in the experimental diets were commonly used in commercial fish feed production. Therefore, no apparent distress in fish was expected by feeding the experimental diets containing black soldier fly larvae. The water quality parameters were maintained at optimal levels and checked frequently during the experiment. In this study no invasive techniques were applied to the fish. No surgery, administration of test substance or physical treatments were performed in live fish. Fish were randomly sampled, anaesthetized, and killed by a sharp blow to the head, in accordance with the Norwegian Animal Welfare act. Skin mucus samples were only retrieved from euthanized fish and other samples were collected after killing the fish.

## Author Contributions

PW, LL, and MØ contributed to conception and design of the study. PW, LL, MS, and JH involved in methodology, investigation, and analysis of data. All the authors contributed to interpretation of data and discussion. LL and MØ acquired funding. PW wrote the first draft of the manuscript. All authors contributed to the article and approved the submitted version.

## Funding

The present study was funded by the Research Council of Norway (RCN), BioTek 2021/Havbruk Biofeed (Grant no. 229003), SureAqua Nordic Center of Excellence (Grant no. 82342) and GutIntraPath (Grant no. 294527).

## Conflict of Interest

The authors declare that the research was conducted in the absence of any commercial or financial relationships that could be construed as a potential conflict of interest.
