# Supplementary material for: Dietary Inclusion of Black Soldier Fly (Hermetia Illucens) Larvae Meal and Paste Improved Gut Health but Had Minor Effects on Skin Mucus Proteome and Immune Response in Atlantic Salmon (Salmo Salar)
Source: Front Immunol. 2021 Feb 25;12:599530. doi: 10.3389/fimmu.2021.599530 (PMC7946862; doi:10.3389/fimmu.2021.599530)
Supplement: Supplementary file 2 [file Table_2.docx]

**Supplementary Table 2.** Unique proteins expressed in the skin mucus of fish fed experimental diets^1^.

| Diet/s | SwissProt accession no | Protein name | Gene name |
| --- | --- | --- | --- |
| Control-1 | A0A1S3L7F1 | Complement C3-like | *LOC106564830* |
|  | A0A1S3L9E0 | Keratin, type I cytoskeletal 18-like | *LOC106565229* |
|  | A0A1S3P8C0 | Mannose-1 phosphate guanyltransferase beta | *gmppb* |
|  | B9EML2 | Tubulin polymerization-promoting protein family member 3 | *TPPP3* |
|  | B5X1Q5 | Serpin H1 precursor | *SERPH* |
|  | B9EM45 | Charged multivesicular body protein 5 | *CHMP5* |
| 6.25IM | A0A1S3MZZ4 | Calumenin isoform X4 | *calu* |
|  | A0A1S2WYQ1 | Secreted phosphoprotein 24 precursor | *spp2* |
|  | A0A1S3KU01 | B-cadherin-like | *LOC106562057* |
|  | A0A1S3M078 | Purine nucleoside phosphorylase | *LOC106569594* |
|  | A0A1S3M8I6 | GTPase IMAP family member 4-like | *LOC106571108* |
|  | A0A1S3N6W3 | Apolipoprotein A-IV-like | *LOC106577505* |
|  | A0A1S3NAU3 | Caspase-7 | *casp7* |
|  | A0A1S3NB18 | Protein disulfide-isomerase A4 | *LOC106578385* |
|  | A0A1S3NRT6 | Alpha-2-macroglobulin-like | *LOC106580921* |
|  | A0A1S3PGC3 | 3-oxoacyl-[acyl-carrier-protein] reductase FabG-like | *LOC106585225* |
|  | B5X3X0 | Serine/threonine protein phosphatase 2A regulatory subunit | *2A5E* |
|  | A0A1S3SWG4 | Heat shock 70 kDa protein 4 isoform X4 | *LOC100380717* |
|  | A0A1S3SCK7 | Calcium/calmodulin-dependent protein kinase type II delta chain isoform X11 | *LOC100380644* |
|  | B5X0R4 | Epidermis-type lipoxygenase 3 | *LOXE3* |
|  | A0A1S3SQT2 | Protein transport protein SEC23 | *LOC106611231* |
|  | B5DG53 | Proteasome subunit alpha type | *psma1* |
|  | B5XFY3 | D-dopachrome decarboxylase | *DOPD* |
|  | B9EP02 | Prothymosin alpha | *PTMA* |
|  | C0HBA2 | Delta-1-pyrroline-5-carboxylate synthase | *P5CS* |
| 12.5IM | A0A1S3LAD7 | Purine nucleoside phosphorylase | *LOC106565393* |
|  | A0A1S3LVJ6 | Septin-2B | *LOC106568816* |
|  | B9EPS7 | RNA-binding protein 4B-like isoform X3 | *RBM4B* |
|  | A0A1S3N9I8 | Putative ferric-chelate reductase 1 isoform X2 | *LOC106578055* |
|  | A0A1S3RMV0 | Serine/arginine-rich splicing factor 7-like isoform X7 | *LOC106603690* |
|  | A0A1S3PUI5 | Calpain-9-like | *LOC106587445* |
|  | A0A1S3Q7I2 | Calpain-2 catalytic subunit-like | *LOC106589985* |
|  | A0A1S3RBE0 | Eukaryotic peptide chain release factor GTP-binding subunit ERF3A | *LOC106601640* |
|  | B5X9W9 | NHP2-like protein 1 | *NH2L1* |
|  | B9EQ25 | High mobility group protein B1 | *HMGB1* |
|  | B5DGS1 | H1 histone family member 0 like protein | *h1f0* |
|  | B5XF34 | Ubiquitin | *UBIQ* |
|  | B9ELC5 | Galectin | *LEG* |
| 25IM | A0A1S3KM47 | Secretory carrier-associated membrane protein | *scamp2* |
|  | A0A1S3KZ47 | Collagen alpha-1(V) chain-like isoform X3 | *LOC106562962* |
|  | A0A1S3LED9 | Protein kinase C | *LOC106565827* |
|  | A0A1S3LPH7 | Neural cell adhesion molecule 1-like isoform X14 | *LOC106567728* |
|  | A0A1S3PY73 | Chromatin target of PRMT1 protein-like isoform X2 | *LOC106588320* |
|  | A0A1S3PZ28 | Chloride intracellular channel protein1-like | *LOC106588213* |
|  | A0A1S3MHB3 | Fibrous sheath CABYR-binding protein-like isoform X4 | *LOC106572634* |
|  | A0A1S3PEW5 | RNA-binding protein EWS-like isoform X6 | *LOC106584976* |
|  | A0A1S3PNB2 | Keratin, type I cytoskeletal 20 isoform X1 | *k1c20* |
|  | B5XCW1 | Peptidylprolyl isomerase | *FKBP3* |
|  | A0A1S3SM10 | Collagen alpha-1 (I) chain-like | *LOC106610502* |
|  | B5XER6 | Sperm acrosome membrane-associated protein 4 precursor | *SACA4* |
|  | B5XGI6 | ARMET | *ARMET* |
|  | C0H894 | Superoxide dismutase | *SODM* |
|  | C0H9R4 | Flotillin-1 | *FLOT1* |
|  | E2JDK6 | Cytochrome c oxidase subunit 2 (Fragment) | *COII* |
| Control-2 | B5DGH4 | ATP synthase subunit b, mitochondrial | *atp5f1* |
|  | A0A1S3RTA3 | C-terminal-binding protein 2 isoform X3 | *LOC106604922* |
|  | A0A1S3QCX2 | Uncharacterized protein LOC106591136 isoform X2 | *LOC106591136* |
|  | B5X1H6 | N-acylneuraminate cytidylyltransferase | *NEUA* |
|  | A0A1S3RX92 | IgGFc-binding protein-like | *LOC106605585* |
|  | A0A1S3S7W2 | Peptidase M20 domain-containing protein 2 | *pm20d2* |
|  | B5DGQ3 | Eukaryotic translation initiation factor 5A | *LOC101448018* |
|  | B5X291 | Disulfide-isomerase A6 | *PDIA6* |
|  | B5XDY6 | Sulfotransferase | *ST1S3* |
| 3.7IP | A0A1S3PUI2 | Toll-interacting protein-like | *LOC106587464* |
|  | A0A1S3L3R8 | Mesothelin-like | *LOC106564109* |
|  | A0A1S3S1Q4 | Uncharacterized protein LOC106606422 | *LOC106606422* |
|  | B5X2E6 | Tripeptidyl-peptidase 1 | *TPP1* |
| 6.7IP | A0A1S3LB33 | SAP domain-containing ribonucleoprotein like isoform X2 | *LOC106565436* |
|  | A0A1S3MEL7 | Procollagen-lysine,2-oxoglutarate 5-dioxygenase 1 isoform X2 | *plod1* |
|  | A0A1S3MXA3 | Bifunctional purine biosynthesis protein PURH-like | *LOC106575709* |
|  | C0H968 | Protein MEMO 1 | *MEMO1* |
|  | A0A1S3QNL7 | C-type lectin lectoxin-Thr1-like | *LOC106594135* |
|  | B5X856 | Calpain small subunit 1 | *CPNS1* |
|  | Q4ZHV0 | Caspase 3B | *CASP3* |
| 6.25IM and 25IM | A0A1S3KKB4 | Fetuin-B-like | *LOC106560587* |
|  | A0A1S3M6D4 | Transcriptional activator protein Pur-beta-like | *LOC106570810* |
|  | A0A1S3M8B3 | Serine/arginine-rich splicing factor 5-like isoform X2 | *LOC106571090* |
|  | A0A1S3M7W8 | Cerebellin-2-like | *LOC106571136* |
|  | A0A1S3NRI0 | Cathepsin K-like | *LOC106580864* |
|  | B5DGY5 | 40S ribosomal protein S3 | *RS3* |
| 6.25IM and 12.5IM | A0A1S3M181 | Calreticulin-like | *LOC106569608* |
| 12.5IM and 25IM | B5XE87 | Protein transport protein Sec61 subunit beta | *SC61B* |
| Control-1, 6.25IM, 12.5IM and 25IM | A0A1S3NSY7 | Fucolectin-6-like isoform X2 | *LOC106581121* |
| Control-1, 6.25IM and 12.5IM | A0A1S3PL84 | Protein-lysine 6-oxidase-like | *LOC106585899* |
|  | A0A1S3RXS9 | Apolipoprotein C-II-like | *LOC106605686* |
|  | B5DH21 | 60S acidic ribosomal protein P1-like |  |
| Control-1, 6.25IM and 25IM | B5XDA4 | Type-4 ice-structuring protein LS-12 | *AFP4* |
| Control-1 and 6.25IM | B5X669 | Charged multivesicular body protein 1b | *CHM1B* |
| Control-1 and 12.5IM | A0A1S3R0V1 | C-type lectin domain family 4 member D-like | *LOC106598857* |
| Control-2, 3.7IP, 6.7IP | B5XCG1 | Programmed cell death protein 5 | *PDCD5* |
| Control-2 and 3.7IP | A0A1S3NAX8 | Phospholipase | *pld1* |
| Control-2 and 6.7IP | B5X5B6 | Protein CutA homolog isoform X2 | *CUTA* |
|  | B9EN79 | Nicotinamide N-methyltransferase | *NNMT* |
| BSFL containing diets | A0A1S3PCH2 | Fibulin-1 | *LOC106584496* |
|  | A0A1S3MH24 | Chromobox protein homolog 5-like | *LOC106572586* |

^1^ Control-1: Control diet. 6.25IM, 12.5IM and 25IM: black soldier fly larvae (BSFL) meal substituted 6.25%, 12.5% and 25% of protein content of Control-1. Control-2: Control diet with 0.88% of formic acid. 3.7IP and 6.7IP: BSFL paste substituted 3.7% and 6.7% of protein content of Control-1.
