# Supplementary figures and images for: Dietary Inclusion of Black Soldier Fly (Hermetia Illucens) Larvae Meal and Paste Improved Gut Health but Had Minor Effects on Skin Mucus Proteome and Immune Response in Atlantic Salmon (Salmo Salar)
Source: Front Immunol. 2021 Feb 25;12:599530. doi: 10.3389/fimmu.2021.599530 (PMC7946862; doi:10.3389/fimmu.2021.599530)

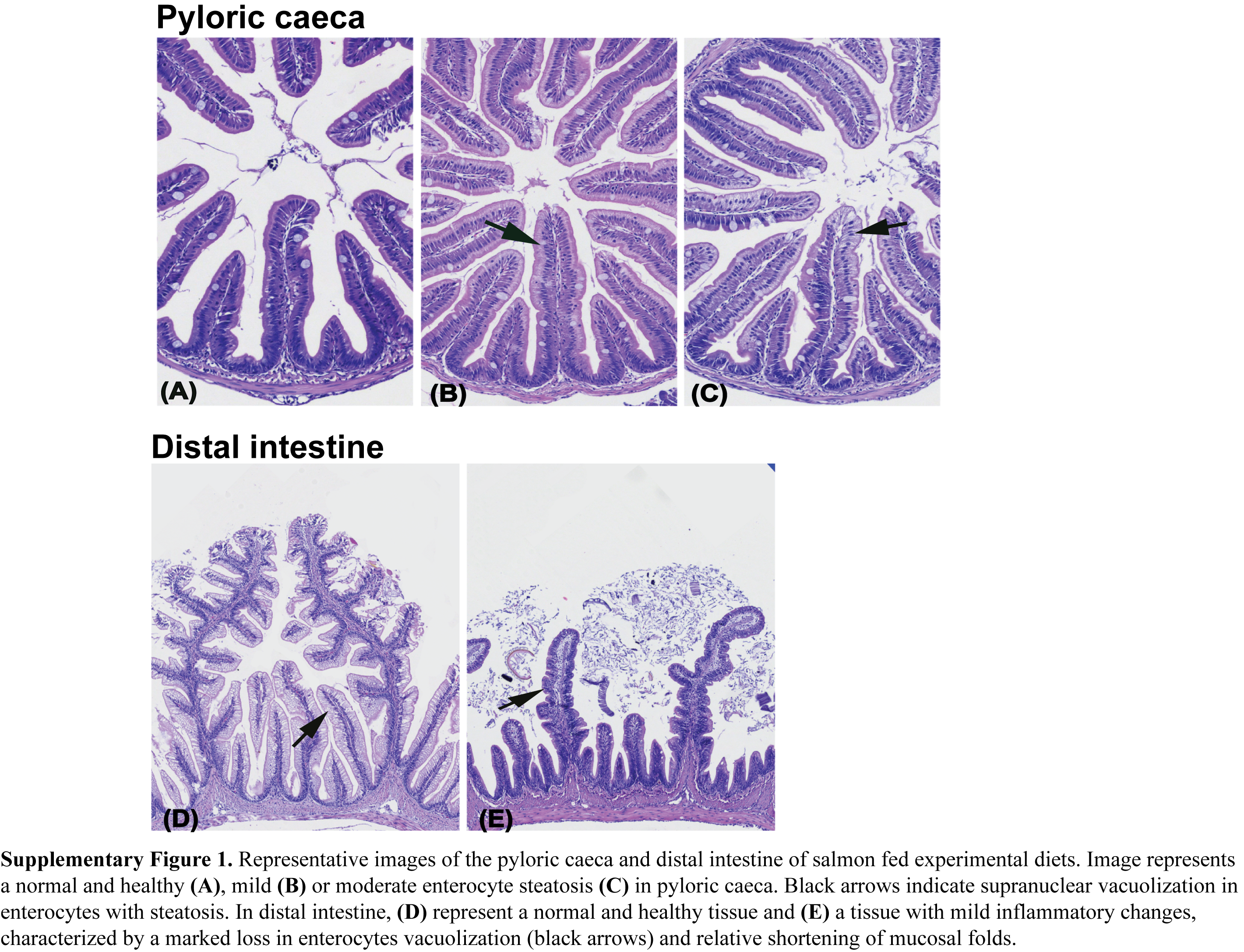

Supplement: Supplementary file 3 [file Image_1.tif]

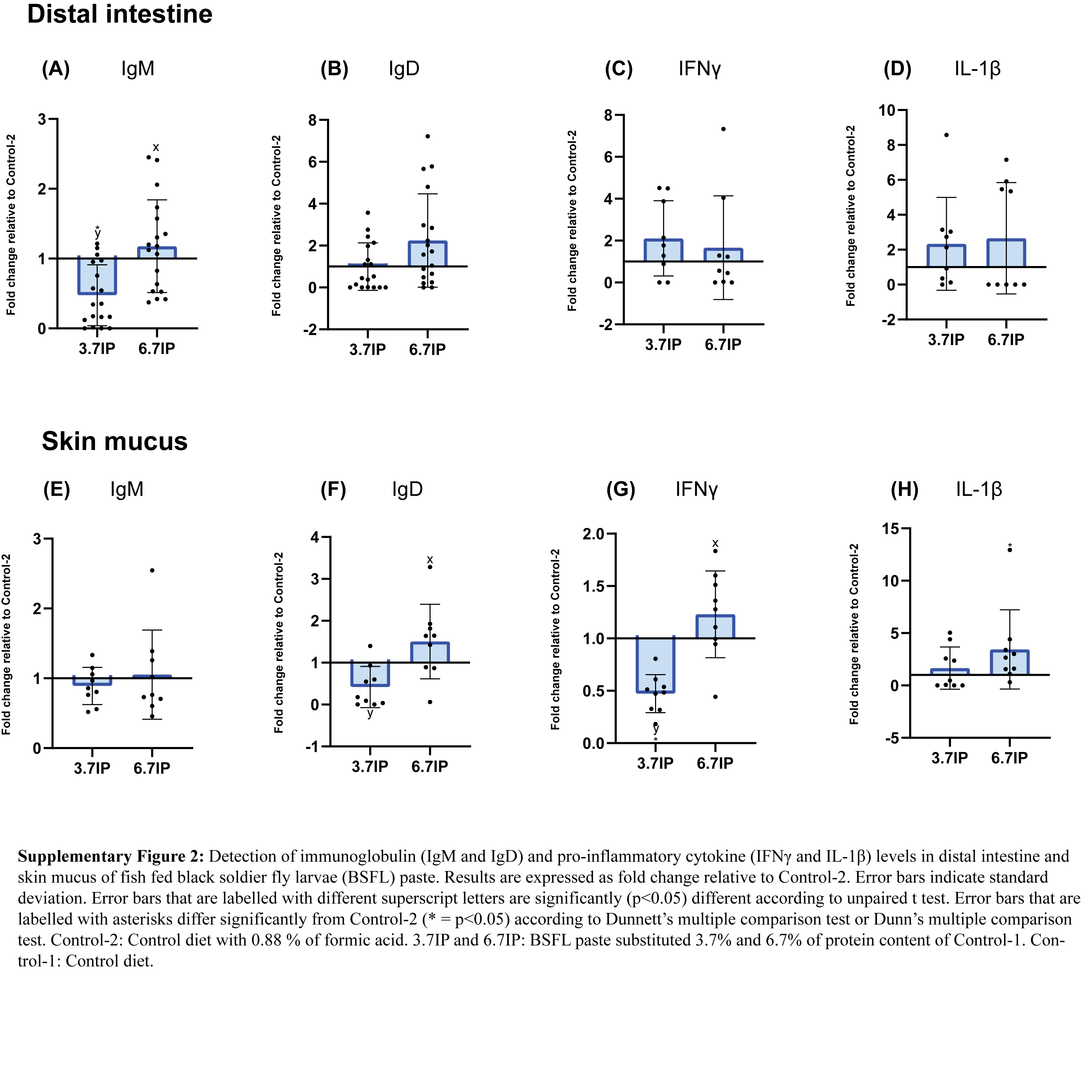

Supplement: Supplementary file 4 [file Image_2.tif]

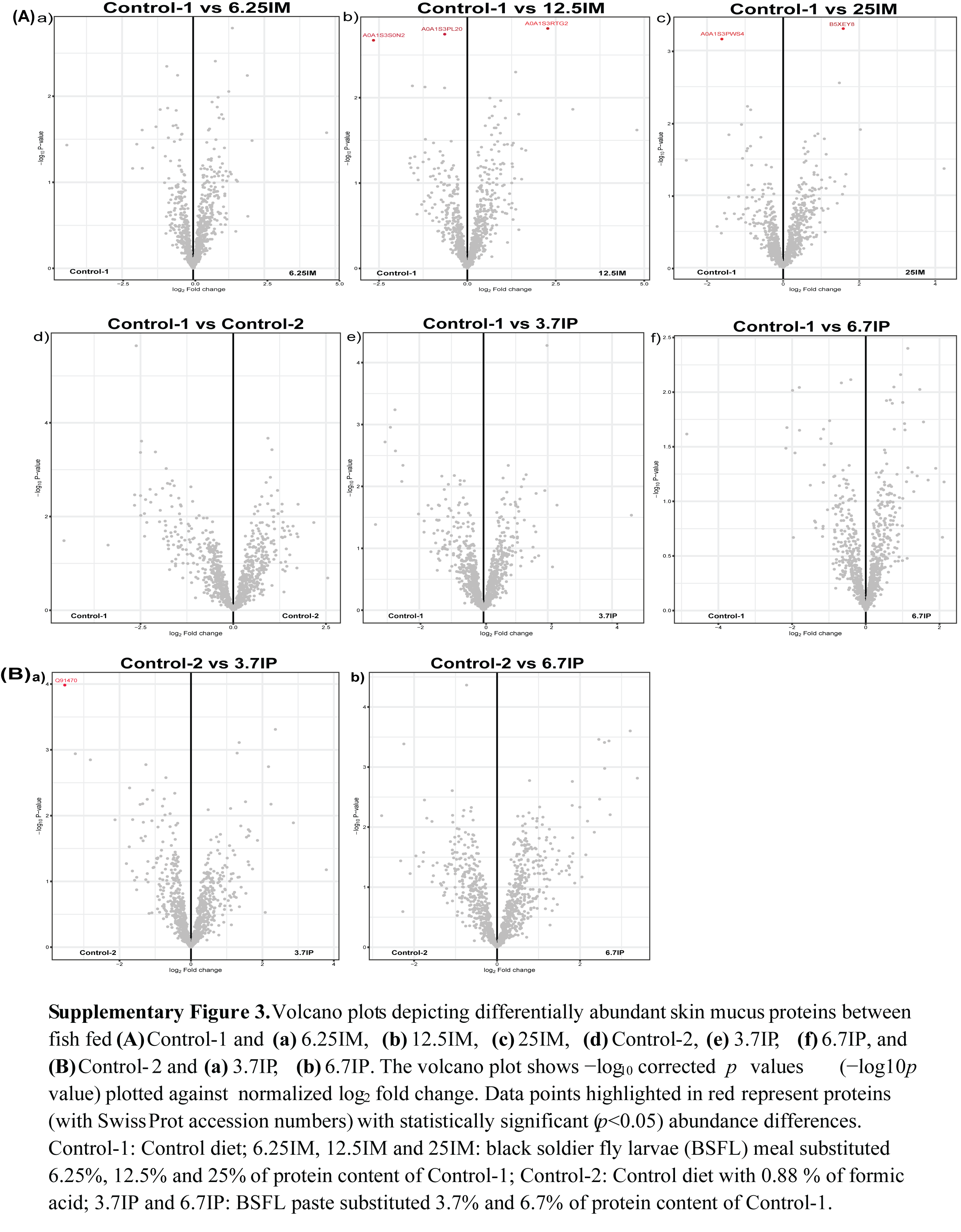

Supplement: Supplementary file 5 [file Image_3.tif]
